# Contribution of Transcranial Direct Current Stimulation on Inhibitory Control to Assess the Neurobiological Aspects of Attention Deficit Hyperactivity Disorder: Randomized Controlled Trial

**DOI:** 10.2196/resprot.4138

**Published:** 2015-05-18

**Authors:** Camila Cosmo, Abrahão Fontes Baptista, Eduardo Pondé de Sena

**Affiliations:** ^1^Spaulding Neuromodulation CenterDepartment of Physical Medicine and RehabilitationSpaulding Rehabilitation Hospital/ Harvard Medical SchoolCharlestown, MAUnited States; ^2^PostGraduate Program - Interactive Process of Organs and SystemsHealth Sciences InstituteFederal University of BahiaSalvadorBrazil; ^3^Functional Electrostimulation LaboratoryBiomorphology DepartmentFederal University of BahiaSalvadorBrazil; ^4^Specialized Support Network CoordinationDepartment of Health (SESAB)Bahia State GovernmentSalvadorBrazil; ^5^PostGraduate Program on Medicine and Human Health,Faculty of MedicineFederal University of BahiaSalvadorBrazil

**Keywords:** Attention deficit hyperactivity disorder, inhibitory control, transcranial direct current stimulation

## Abstract

**Background:**

The applicability of transcranial direct current stimulation (tDCS) in individuals with attention deficit hyperactivity disorder (ADHD) has not yet been investigated. This low-cost, non-invasive, and safe technique optimized to modulate the inhibitory response might be a useful treatment option for those affected by this condition.

**Objective:**

The aim of this single center, parallel, randomized, double-blinded, sham-controlled trial is to investigate the efficacy of transcranial direct current stimulation over the prefrontal cortex on the modulation of inhibitory control in adults with attention deficit hyperactivity disorder.

**Methods:**

A total of 60 individuals will be divided into 2 groups by block randomization to receive active or sham stimulation. Anodal stimulation over the left dorsolateral prefrontal cortex will be applied at 1 mA during a single 20-minute session. Before and after interventions, subjects will perform 2 go/no go tasks and the brain electrical activity will be recorded by electroencephalogram (EEG) with 32 channels, according to the 10-20 international EEG system.

**Results:**

The trial began in May 2013 and we are currently performing the statistical analysis for the secondary outcomes.

**Conclusions:**

The findings from this study will provide preliminary results about the role of prefrontal cortex activation through tDCS on ADHD patients.

**Trial Registration:**

Clinicaltrials.gov NCT01968512; http://clinicaltrials.gov/ct2/show/NCT01968512 (Archived by WebCite at www.webcitation.org/6YMSW2tkD).

## Introduction

Attention deficit hyperactivity disorder (ADHD) presents prevalence rates in US studies of 5%-9% in childhood, especially in school age children. Of those affected, 67% continue to present symptoms in adulthood, which may compromise psychosocial, professional, and emotional development [1-5]. In a recent study, Arruda et al observed a prevalence rate of ADHD among children and adolescents of 4.4% [6]. However, despite the significant social impact, some of these patients remain undiagnosed and therefore without proper therapeutic intervention. The diagnosis, based on clinical assessment and identification of symptoms established by the Diagnostic and Statistical Manual of Mental Disorders, Fourth Edition, Text Revision (DSM-IV-TR), involves inattention, hyperactivity, impulsivity, and general criteria [7,8].

The clinical presentation of ADHD may arise from disorders in the prefrontal cortex and its subcortical connections [8-11]. This assumption is supported by the hypothesis that it is a neurobiological disorder, evidenced by functional deficits in neurotransmitters such as dopamine and norepinephrine, and by disorders in the frontal lobe. These disorders trigger the symptoms observed in ADHD patients including impulsivity, difficulty in inhibiting distracting behaviors, and difficulty in planning and executing tasks under focused attention and concentration [10,12]. Together, these symptoms reveal the impairment of important executive functions in individuals with ADHD [1,2,9,13].

There is evidence of a physiological association between the prefrontal cortex and inhibitory control [9,14], whereby changes in this anatomical structure may account for impairments in inhibitory control. Indeed, behavior-based actions that reflect inconsequence, unpredictability, intolerance to waiting, quick and inconsistent responses, possible exposure to hazards, and acceptance of multiple responsibilities and tasks simultaneously with consequently further withdrawals support this idea [9].This anatomical relationship between prefrontal cortex and inhibitory behavior, together with an understanding of the neurobiological mechanisms comprise a physiological framework that supports the hypothesis that activation of the prefrontal area will result in a larger and more suitable inhibitory control, minimizing the socioaffective consequences associated with this condition.

Transcranial direct current stimulation (tDCS) is a simple and well-known neuromodulatory technique [15,16] which involves applying low voltage electrical currents to increase or decrease neuronal excitability of the stimulated area [16,17]. tDCS is a noninvasive and safe approach, characterized by infrequent and mild adverse effects including local discomfort, itching, paresthesia, and/or short-lasting headaches [17].

The aim of this trial is to investigate the efficacy of tDCS over the prefrontal cortex on the modulation of inhibitory control in adults with ADHD by assessing behavioral and neurophysiological parameters. We expect that the application of this low-cost and safe technique will increase the inhibitory response in ADHD individuals what may result in a greater ability to inhibit inappropriate behaviors.

## Methods

### Study Design

This is a single-center, parallel, randomized, double-blinded, sham-controlled trial, conducted in the Laboratory of Functional Electrostimulation at the Federal University of Bahia (Salvador, Brazil). This study will include descriptive and analytical steps designed to investigate the neurobiological aspects of patients with ADHD, and the response of inhibitory control parameters to tDCS.

### Population

Patients with ADHD will be recruited by sending letters and emails to neuropsychiatric societies and associations. Professional experts in ADHD, contacted by mail and telephone, will be invited to refer potential patients for the study. Advertisements will be published on the Internet and social networking sites, according to the inclusion criteria.

### Inclusion Criteria

The inclusion criteria for the study are (1) the ability to understand and sign the informed consent form, (2) a diagnosis of ADHD according to DSM-IV-TR and the Adult Self-Report Scale (ASRS), (3) resident of Bahia, and (4) ≥18 years old.

### Exclusion Criteria

Patients who meet the following criteria will be excluded (1) major psychiatric disorders such as schizophrenia and bipolar disorder, (2) inability to understand the questionnaires applied (cognitive impairment score of ≤24 on the Mini-Mental State Examination) or illiterate, and (3) abuse of psychoactive substances or alcohol, except nicotine and caffeine, during the last 12 months.

### Sample Size

A sample size of 50 subjects was calculated assuming a difference in proportion between the active tDCS and sham control groups of 40% on the go/no go performance, before and after interventions. This calculation was performed using the statistical program STATA 12.0, considering an alpha of .05 and a power of 0.80, resulting in a sample of 25 subjects in each group (intervention and control). To address unexpected factors, we applied a dropout rate of 20%, reaching a total sample size of 60 individuals.

### Randomization and Blinding

Individuals will be divided into two groups by block randomization aiming for a balanced distribution between the groups (30 subjects each), and considering gender and age as variables. Blocks will be composed of 10 subjects, totaling 6 blocks. Each intervention group will comprise 5 individuals from each block.

An external researcher will perform the randomization and will generate a list to allocate patients and ensure a concealed allocation.

Excluding the external researcher, investigators applying the go/no go tasks and registering the EEG, and the subjects will be blind to the intervention. Raters and researchers responsible for the statistical analysis will not be aware of the treatment group that the patient will be enrolled. For analysis purposes, the intervention groups will be identified as “0” and “1”. Blinding code will be known only by the principal investigator and by the person responsible for the stimulation.

### Ethical Aspects

In accordance with the Declaration of Helsinki [18], this study strictly follows the ethical principles in research involving human subjects. All participants will be informed about the nature of the study and all procedures prior to enrollment. Following the resolution 196/96 of the National Health Council (Brazil), only those that will sign the informed consent form will be included. The signature of a witness will be required for the patients unable to sign the free and informed consent form.

This trial was approved on October 2012 (IRB approval number 19311) by the institutional review board of the Maternidade Climério de Oliveira-Federal University of Bahia.

### Consent Procedures

Signed consent will be obtained from each participant. Only subjects capable of understanding and agreeing to the consent will be enrolled. In addition to a signed consent form and prior to any intervention, participants will receive a detailed explanation about the trial aims and procedures that will be performed. Two experienced researchers will explain that participation is voluntary and they may withdraw at anytime without losses. The trial staff also will be available to clarify any question; at the same time they will assure that subjects understood all of the steps of the study. Participants will have an indefinite amount of time to make a decision, and those that agree, will be asked to sign two copies of the informed consent form.

### Assessments

Following the consent procedures, subjects will undergo a cognitive screening using the MiniMental State Examination (MMSE) [19,20], the Mini International Neuropsychiatric Interview Brazilian (MINI PLUS) [21], the Adult Self-Report Scale (ASRS) [22], the Adult ADHD Quality of Life Questionnaire (AAQoL), and an interview with a questionnaire. This evaluation script is an instrument based on the ADHD diagnostic criteria from DSM-IV-TR [7,8], and consists of epidemiological and clinical questions.

Posterior electrical brain activity will be recorded by EEG with 32 channels placed on the scalp, according to the 10-20 international EEG system. Each recording will last 5 minutes and will be performed in a resting state, with the patient's eyes open and looking for a fixed point during the first minute, and with closed eyes for the remaining four. Participants will then be asked to perform tasks on the computer: 2 go/no go tasks adapted from the original version using a fruit and a letter as the first and second target, respectively. Following the cognitive tasks, subjects will undergo the active or sham stimulation, according to the previous randomization. At the end of the intervention, subjects will perform the 2 go/no go tasks again (randomly selected to avoid learning effect), and they will undergo another EEG recording.

There will be no follow-up visits; participants will undergo a single visit of approximately two hours in duration. All subjects will be asked to abstain from caffeine, alcohol, and nicotine one day before the experiments.

### Interventions

Participants of the intervention group will have the tDCS applied at 1 mA, with the anode electrode over the left dorsolateral prefrontal cortex and cathode electrode over the equivalent area on the right side. For the control group subjects, electrodes will be placed at identical positions and sham stimulation will be applied. The stimulation device will be turned on for 30 seconds so that the patient will feel the initial sensation, then will be shut down. The intervention and sham procedure will take place in one single 20-minute session to evaluate the immediate effect.

The application of tDCS and the assessment of its effects will be performed by clinical observation using a reduction of symptomatology as evaluated by the neuropsychological task, go/no go, and by neurophysiological assessment, measured by quantitative electroencephalogram (qEEG) and the reconstruction of brain networks, as parameters.

### Outcome Variables

The primary outcome of this trial is the inhibitory response measured as behavioral performance by two go/no go tasks. These tests are adapted from a previous version [23], and will present as targets a fruit (version 1) and a letter (version 2). Participants will be asked to react when a target ("go" stimulus), and not when a non-target ("no go" stimulus) is presented. All stimuli will be presented in black and white ink with equal dimensions. In both versions of the go/no go tasks, one selected letter or fruit will be previously defined as the "go" stimulus and subjects will be instructed to press the left button of a computer mouse with the right finger as soon as this target is presented on the screen. Stimuli will be presented one-by-one on the computer screen during 650 milliseconds with 1000 millisecond interstimulus intervals (ISI). In each version of the task, 150 trials consisting of 80% "go" and 20% "no go" will be presented. Correct responses, impulsivity, and omissions errors will be computed separately for each version of the go/no go task, and will be the primary outcome variables.

The secondary outcome variables are power analysis of frequency bands through qEEG and the brain networks. For the EEG, data will be recorded from the 32 channels and a reference electrode (Cz) placed according to the 10-20 international EEG system with additional electrodes (FC3, FC4, CP3, CP4, FT7, FT8, TP7, TP8, and Oz). The EEG data will be filtered between 0.5-50 Hz, and analyzed through EEGLAB (The Mathworks, Inc.). Brain network reconstructions will be performed based on the EEG data.

### Statistical Analysis

A 5-step statistical analysis will be conducted (see Textbox 1). These analyses will be performed using Stata, version 13.0 for Windows. Results will be considered statistically significant if *P*<.05.

**The 5-step statistical analysis.**

Steps

1. The socio-demographic, clinical, and epidemiological description of the groups, using the usual procedures of descriptive statistics such as calculation of frequencies, measures of central tendency, and dispersion;
2. Characteristics will be compared between groups at baseline, using a one-way analysis of variance (ANOVA) for continuous variables and a chi-square test for categorical variables;
3. Shapiro-Wilk test will be performed to assess the normality assumption of the outcome variables;
4. Analysis of paired and independent samples using a *t* test for comparisons within each group and between intervention groups, or applying equivalent non-parametric tests (according to Shapiro-Wilk test results) before and after intervention;
5. Pearson chi-square test to evaluate blinding effectiveness, comparing between groups.

## Results

This trial and enrollment began in May, 2013. The statistical analysis for the secondary outcomes is currently being performed.

## Discussion

The findings from this trial will provide preliminary results about the role of prefrontal cortex activation through tDCS on ADHD patients. The results of this clinical trial will allow us to evaluate behavioral and neurobiological aspects of ADHD in addition to observing the socio-demographical and clinical-epidemiological parameters of this population.

We will examine the tDCS contribution in modulating inhibitory control and neurophysiological parameters measured by neurocognitive tasks (go/no go), qEEG, and brain reconstruction network models. We also expect that the present trial will contribute scientifically to the development of neurophysiological assessment methods of ADHD, and to the evaluation of the feasibility of this low cost, non-invasive, and safe technique for optimization of inhibitory responses in ADHD patients.

One possible limitation is that we will only apply a single session of tDCS. However, since, to the present moment, there are no previous studies regarding tDCS in ADHD subjects, we opted for a conservative protocol with only one session of tDCS delivered at 1 mA to ensure safe parameters.

To the best of our knowledge, this trial will be the first study to assess the cognitive and neurophysiological effects of tDCS on ADHD patients. In the long term, we expect that our results might reinforce comprehensive programs of intervention and multidisciplinary approaches in patients with ADHD.

## Abbreviations

AAQoL: Adults with ADHD Quality of Life Questionnaire
ADHD: Attention deficit hyperactivity disorder
ASRS: Adult Self-Report Scale
DSM-IV-TR: Diagnostic and Statistical Manual of Mental Disorders, Fourth Edition, Text Revision
EEG: Electroencephalogram
ICF: Informed consent form
MINI-plus: Mini International Neuropsychiatric Interview Plus
MMSE: Mini Mental State Examination
qEEG: Quantitative electroencephalogram
tDCS: Transcranial direct current stimulation
